# Extracellular Vesicles & Co.: scaring immune cells in the TME since ever

**DOI:** 10.3389/fimmu.2024.1451003

**Published:** 2024-08-29

**Authors:** Carlo Rodolfo, Silvia Campello

**Affiliations:** Department of Biology, University of Rome Tor Vergata, Rome, Italy

**Keywords:** extracellular vesicles (EVs), migrasomes, exosomes, TME (tumor microenvironment), autophagy

## Abstract

The health tissue surrounding a solid tumor, namely the tumor microenvironment (TME), is an extremely complex universe of cells, extracellular matrix, and signals of various nature, that support and protect the growth of cancer cells. The interactions taking place between cancer cells and the TME are crucial not only for tumor growth, invasion, and metastasis but they also play a key role in modulating immune system responses to cancer, and vice-versa. Indeed, tumor-infiltrating immune cells (e.g., T lymphocytes and natural killers) activity is greatly affected by signals (mostly ligands/receptors and paracrine) they receive in the TME, which frequently generate an immunosuppressive milieu. In the last years, it has become evident that soluble and receptor signaling is not the only way of communication between cells in the TME, with extracellular vesicles, such as exosomes, playing a central role. Among the different new kind of vesicles recently discovered, migrasomes look like to be of extreme interest as they are not only different from the others, but also have been reported as able to deliver a very heterogeneous kind of messages, able to profoundly affect recipient cells’ behavior. Indeed, the role played by the different classes of extracellular vesicles, especially in the TME, relies on their not-directional diffusion from the originating cells, while migrasomes released from migrating cells do have a directional effect. Migrasomes biology and their involvement in cancer progression, dissemination, and resistance to therapy is still a largely obscure field, but with promising development foreseen in the next future.

## Tumor microenvironment

The tumor microenvironment is a complex region comprising structural components and different types of cells, such as mesenchymal-derived cells, resident and/or infiltrating vessels (endothelium), and cells of both innate and adaptive immunity, including lymphocytes, natural killer cells (NKs), dendritic cells (DCs), and macrophages. All these cells talk to each other in the TME by means of contact (cell-cell and cell-matrix interactions), soluble (ligands-receptors), and vesicle-delivered signals. The complex cell signaling taking place in the TME plays a key role and the “tumor microenvironment is not just a silent bystander, but rather an active promoter of cancer progression” (1), with resident cells able to modulate immune system activity against the tumor itself (2, 3). Indeed, tumor-infiltrating T lymphocytes (TILs) activity and efficacy are strongly affected by the balance between cellular and humoral components, as well as by the diverse inflammatory responses in the TME region, a crosstalk that frequently results in the establishment of an immunosuppressive milieu. Tumor cells often adopt mechanisms to hide their vulnerability by exploiting physiological immune checkpoints, such as those based on PD1/PD-L1 and CTLA4, to limit immune cell activation, and “metabolic checkpoints” (e.g., mTOR, PGC-1a), to win the competition for nutrients and metabolites. On balance, infiltrating immune cells undergo exhaustion and fail to kill the cancer cells. TME’s immunosuppressive nature is the major hurdle the immune system must overcome, to be able to attack the developing tumor, and impacts on the efficacy of new immunotherapy approaches, such as CAR-T (4).

The understanding of the complex signaling network established between cancer cells and TME has greatly improved. Nevertheless, there is not yet a comprehensive picture about the contribution of players other than the immune cell receptors/ligands and soluble (cytokine, chemokines) signaling as well as on the interplay between “classic” and “new” players. In this context, extracellular vesicles (EVs), released both from cancer and other TME-resident cells, have been shown to act as mediators of immune system activation (5), as well as to sustain cancer growth and dissemination (6, 7). EVs are cell-derived membrane-surrounded vesicles of various size, originated/released by different kind of cells in the TME, and able to carry various messages to other cells, for a detailed review see (8).

The role played by EVs in the TME is far to be completely unveiled, and in the last decade a new class of EVs, named migrasomes (9, 10), and new roles for already known EVs, such as apoptotic vesicles (ApoEVs, (11), have been identified.

## Extracellular vesicles & Co.

In the last years, more and more complexity has been added to “the extracellular vesicles club”, mainly because of “new members” identification, newly described functions for “old members”, and the identification of non-vesicular extracellular matter. Indeed, exosomes, microvesicles, and apoptotic bodies, once considered as classical EVs, where recently joined by autophagic, stressed (12), and matrix vesicles (13), as well as oncosomes (14), migrasomes (9), and nanoparticles (15). This heterogeneity prompted to a revisitation of the classification criteria, by taking in consideration different key aspects, such as size, biogenesis, and originating cell (see **Table 1**), and opened a debate on the role played by these different vesicles and their contents as well as on their possible exploitation, as diagnostic markers, or therapeutic strategy.

**Table 1:** Extracellular Vesicles (EVs) and Non-vesicular Extracellular Nanoparticles (NVEPs) characteristic.

Category	Name	EV Class	Size	Membrane	Markers	Biogenesis	Diffusion
**Exosomes**	Classical exosomes	Small	40-150 nm	Lipid bilayer	Alix, Tsg101, CD63, CD9, CD81	Multivesicular endosome	No fixed direction
	Non-classical exosomes			Lipid bilayer	Alix, Tsg101, CD63/CD9/CD81 negative		
**Microvesicles**	Classical microvesicles	Large	150-1000 nm	Lipid bilayer	Annexin A1, ARF6	Plasma membrane shedding	No fixed direction
	Large oncosomes		1-10 μm	Lipid bilayer	Annexin A1, ARF6		
	ARMMs	Small	40-100 nm	Lipid bilayer	ARRDC1, TSG101		
**Migrasomes**	Migrasomes	Large	0.5-3 µm	Lipid bilayer	NDST1, CPQ, PIGK, EOGT	Before left cell, migrasomes are organelles	Along the migratory path
**Apoptotic EV**	Apoptotic bodies	Large	1-5 μm	Lipid bilayer	Annexin V, PS	Apoptosis	No fixed direction
	Apoptotic vesicles	Small to Large	100-1000 nm	Lipid bilayer			
**Autophagic EV**	Autophagic EV	Small to Large	40-1000 nm?	Lipid bilayer	LC3B-PE, p62, dsDNA/Histones	Autophagosome- endosome fusion (Amphisome)	No fixed direction
**Stressed EV (Stressome)**	Stressed EV Damaged EV	Small to Large	40-1000 nm?	Lipid bilayer	HSP90, HSPs	Plasma membrane shedding, autophagy	No fixed direction
**Mitochondrial extracellular vesicles**	Mitochondrial-derived vesicles	Small	70–150	Lipid bilayer	Mitochondrial proteins	Mitochondria	No fixed direction
	Mitovesicles	Small to Large	50–350 nm	Single to triple lipid bilayer	VDAC, COX-IV, PDH-E1α		
**Matrix vesicles**	Matrix vesicles	Small to Large	40-1000 nm?		Fibronectin, Proteoglycans	Matrix binding and release	No fixed direction
**Non-vesicular extracellular nanoparticles**	Exomeres	Non-EV	30-50 nm	No	TGFBI, HSPA13, ENO2	Unknown	No fixed direction
	Supermeres	Non-EV	20-30 nm	No	FASN, ACLY	Unknown	No fixed direction

For a complete review see (10, 15, 16).

### Exosomes

Exosomes, probably the most studied and characterized among EVs, are small (40-150 nm Ø) vesicles produced in the endosomal compartment of most eukaryotic cells, during the maturation of multivesicular endosomes (MVEs), secreted upon MVEs fusion with the cell surface, and have specialized functions in different physiological processes (10, 17). Nearly all exosomes, independently from the cell type of origin, share proteins related to endosome maturation (e.g., Annexins) and tetraspanins, which can be used as surface markers. Exosomes’ activity as intercellular communicators rely on both their membrane composition, in terms of lipids and receptors, as well as on their cargo. Both these characteristics are strictly related to the cell of origin, and allowed the delivery of different messages, by means of specific combinations of functional molecules, lipids, proteins (both native and aggregated), DNA, and RNA (mRNA, microRNA, and other non-coding RNA). Once internalized, these cargoes can actively change recipient cells’ habits as well as severely impact the nature of the environments where this exchange happens. In this scenario, the information’s exchange happening through exosomes in the TME is of extreme importance not only for the resulting alteration of the stromal cell phenotypes around the tumor, able to promote progression and metastasis, but also for the messages tumor cells deliver to the immune ones, to limit their effectiveness.

### Microvesicles

Microvesicles share structural similarity with exosomes, but differ in size, lipid composition, content, and above all origin, as they generate by shedding of the plasma membrane micro-domains, such as lipid rafts or caveolae (18). Nevertheless, their cargo is selectively recruited (19) and comprises membrane-derived receptors, proteins, lipids, carbohydrates, and genetic material such as mRNA and microRNAs. Its composition depends upon the parent cell, the microenvironment, and the specific release-triggers, and can be released, thus altering the extracellular milieu, or internalized by recipient cells, thus impacting on their activity. Microvesicles can be classified as small (40-100 nm), such as arrestin domain containing protein 1 (ARDCC1) mediated microvesicles (ARMMs), which form by a budding virus-like mechanism (20); or large (0.1-10 µm), such as classical microvesicles and large oncosomes. Classical microvesicles were first identified in the circulatory system as able to coordinate pro-coagulatory and inflammatory response (21), and are now recognized as released by almost all kind of cells (22). Tumor-derived MVs can inhibit tumor response to immunotherapy but they can also be exploited as cancer biomarkers (in body fluids), to reverse cancer cells’ drug resistance, to deliver chemotherapeutic drugs and oncolytic adenoviruses (23). Large oncosomes, are cancer cell-derived EVs able to deliver tumor-promoting molecules and to induce transformation in the recipient cells (24). The number of large oncosomes released by a certain cancer cell is directly correlated with its aggressiveness as they can alter TME homeostasis by acting on both TME resident cells and extracellular matrix structure and composition (25).

### Apoptotic, autophagic, and stressed EVs

Stressed or dying cells can release EVs, with defined content and role in cell-to-cell communication. Indeed, cells dying by apoptosis release a variety of EVs, known as apoptotic cell derived EVs (ApoEVs, 100-5000 nm Ø), playing a role in many aspects of immunity and disease, by either activating or dampening immune responses (26). Autophagic EVs (AEVs, 40-1000 nm Ø) were recently identified as released by fusion of amphisomes with the plasma membrane. Indeed, autophagosome generated during autophagy induction can either fuse with the lysosome, to allow cargo degradation, or with endosomes, to give rise to a hybrid organelle termed amphisome, which then fuse with the plasma membrane to release AEVs (27). Both ApoEVs and AEVs could be related to amphisome formation, thus suggesting a more general mechanism for diverse cellular components. Stressed cell-derived EVs are part of the so-called “stressome”, a term used to identify cell stress-induced secretion products and EVs (12).

### Migrasomes

Migrasomes are large (0.5-3 µm) EVs, with two peculiar features not presented by other EVs: prior to release they are organelles, and they are directionally released. In fact, migrasome grow at the intersections or tips of retracting fibers and are released at the back of migrating cells through retraction fibers (9, 28). Migrasome cargo comprises different cellular components such as lipids, proteins, RNA, as well as organelles. Once taken up by other cells, migrasome can rapidly deliver information, thus regulating physiological processes, such as embryonic development, and tumor invasion or migration (29–31). The formation and release of migrasomes relies on the rearrangement of both the cytoskeleton and the mitochondrial network, as well as on the activity of the Tetraspanin-4 (TSPAN4) protein at the membrane (28, 32, 33).

### Mitochondria derived extracellular vesicles

Mitochondria derived extracellular vesicles (MitoEVs) are a heterogenous class of EVs, originating from the release of Mitochondrial-derived vesicles (MDVs, ∼70–150 nm), via the MVB or microvesicle mediated pathways (16). Recently, mitovesicles (50–350 nm) have been identified as a specific subset of EVs, containing mitochondrial derived material (34) in brain tissues. It has been proposed that mitochondrial dynamics might somehow play a role in the generation of MDVs and mitovesicles and that through the release of mitochondrial contents cells aim to maintain their own homeostasis and eventually impact on the functionality of recipient cells.

### Matrix vesicles

Matrix vesicles are mainly produced by cancer-associated fibroblasts and cancer cells and comprise both extracellular matrix-bound vesicles, embedded in the extracellular matrix (ECM) of tumours’ stroma, and matrix-coated vesicles, in the TME (35). Matrix-coated vesicles/ECM interaction can lead to tumor-infiltrating immune cells reprogramming, thus impacting tumors’ progression and dissemination.

### Non-vesicular extracellular nanoparticles

Non-vesicular extracellular nano particles (NVEPs), are characterized by the absence of a lipid bilayer membrane, include well-known entities, such as lipoprotein particles and nucleosomes, but also the recently discovered exomeres and supermeres. Exomeres have been identified as a type of small (<50 nm), non-membranous nanoparticle, carrying a cargo enriched with proteins implicated in regulating metabolic pathways, such as glycolysis and mTORC1, and specifically associated with ER, mitochondria, and microtubules, so suggesting a potential implication of these organelles in their biogenesis and secretion (36–38), which are still quite obscure. Supermeres, named as they are the supernatant of exomeres pellet after isolation, are thus smaller than exomeres and exhibit selective enrichment of proteins and especially extracellular RNA (39, 40). As of today, it cannot be excluded that exomeres and supermeres derive from a larger overlapping population of NVEPs (15).

## EVs and TME

**Figure 1:**
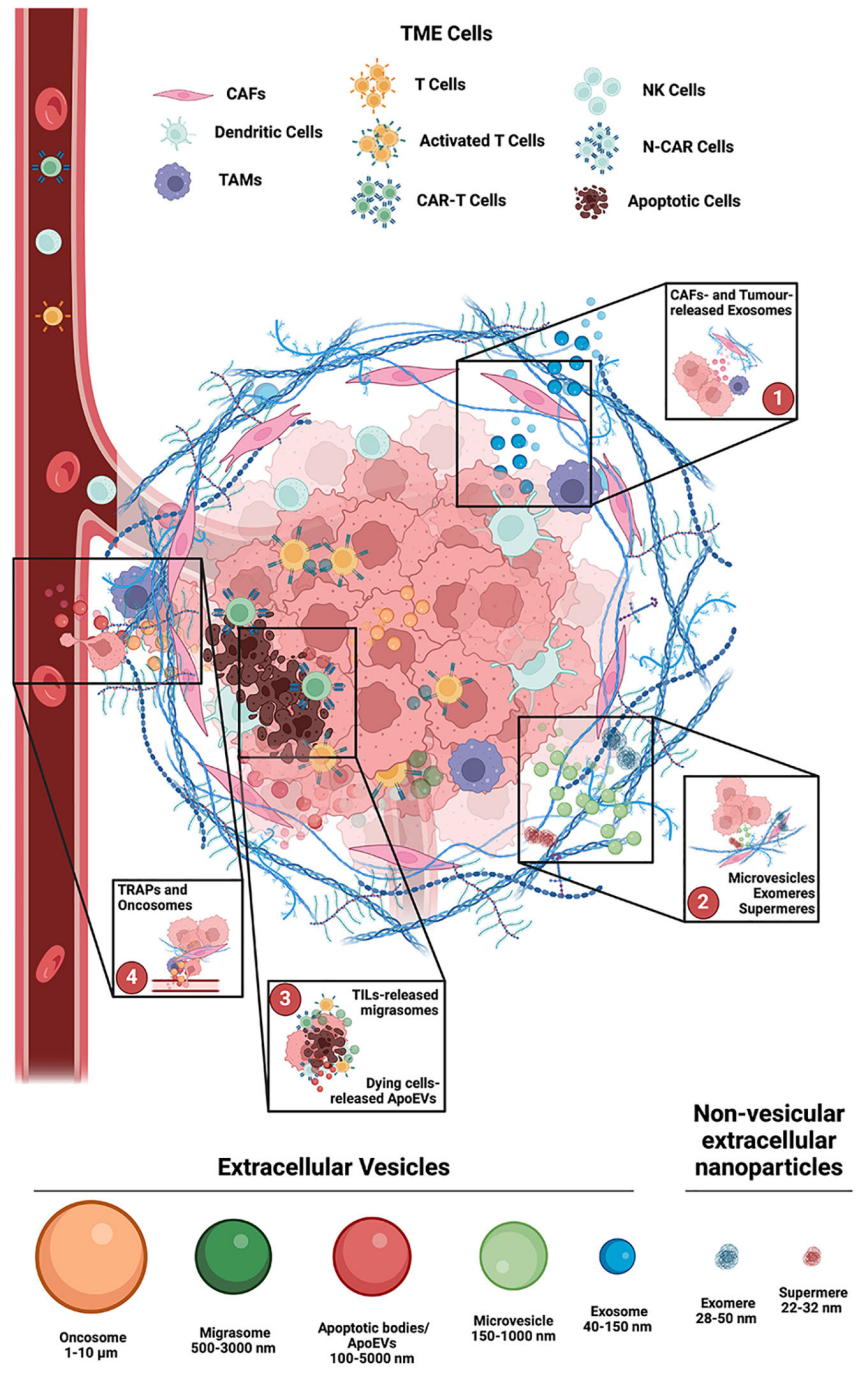
Main involvement of Extracellular Vesicles in the TME. 1. Exosomes (40-150 nm Ø) are produced in the endosomal compartment of both Cancer Associated Fibroblasts (CAFs) and Tumor cells. Lipids and receptors in their membrane composition, as well as their cargo, are strictly dependent upon the cell of origin, and in the TME they can induce stromal cells’ phenotype changes, to promote tumors’ progression and metastasis, as well as to limit immune cells effectiveness. 2. Microvesicles (150-1000 nm Ø) are like exosomes, but differ in size and origin, as they generate by shedding of the plasma membrane micro-domains. Their selectively recruited cargo depends upon the parent cell, the microenvironment, and the specific release-triggers. Non-vesicular extracellular nano particles (NVEPs), such as exomeres and supermeres, are characterized by the absence of a lipid bilayer membrane. 3. Migrasomes (500-3000 nm Ø) have two unique features: prior to release they are organelles, and they are directionally released, at the back of migrating cells, like tumor infiltrating lymphocytes (TILs). Apoptotic cell derived EVs (ApoEVs, 100-5000 nm Ø) and Autophagic EVs (AEVs, 40-1000 nm Ø) are released, respectively, during cell death and cell stress. 4. Tumor cell-released autophagosomes (TRAPs) and large oncosomes (1-10 µm Ø), can induce recipient cells transformation, and alter TME homeostasis by acting on both structure and composition of TME resident cells and of the extracellular matrix. Created with BioRender.com.

Tumor progression not only relies on cancer cells’ unregulated growth but also on the interactions they establish with the surrounding microenvironment. TME is a very complex region comprising various cell types and conditions (see above and **Figure 1**). In this peculiar environment, cancer cells are continuously exposed to different sources of stress, such as hypoxia, acidity (pH), oxidative, mechanical, thermic, nutrient, genotoxic, etc. To cope with this critical situation, cancer cells can produce and release EVs, as a mean to impact on TME resident cells as to create a favorable niche for their survival and propagation. All the EVs described above have the potential to deliver messages favoring tumor cells’ survival, i.e. by impacting on immune cells’ activity in the TME or by establishing chemoresistance (8). Indeed, from an immunological point of view, tumors can be defined as: “hot tumors”, in which CD8^+^ T cells infiltrate the tumor mass and their activity can be fostered by means of immune checkpoint inhibitors (ICI); and “cold tumors”, that completely excluded or deserted T cells, and thus are ICI resistant (41). In this scenario, the soluble signaling relying on tumor cells’ secreted molecules, such as cytokines, is further sustained by the release of EVs. Exosomes released by tumor cells could deliver inhibitory molecules, such as PD-L1, able to neutralize ICI and PD1^+^ T cells. Moreover, in response to monoclonal antibody or chemotherapy, cancer cells, fibroblasts and resident immune cells can release different type of EVs containing specific cargoes able to induce chemoresistance.

The above described EV effects are just simple examples of EVs activity in the TME, where they are essential for establishing the “cold tumors” phenotype, mainly by: i) promoting angiogenesis and extravasation; ii) promoting immunosuppression, by inducing immune cells apoptosis or disabling NK cells; iii) changing the differentiation or polarity of various cell types into pro-tumorigenic, immunosuppressive, anti-inflammatory, and chemoresistant phenotypes (42).

## Mitochondria, migrasomes and TME signaling

Mitochondria and mitochondrial dynamics (mito-dynamics) are of extreme importance for tumor development as well as for the recruitment and activity of immune cells within the TME (43). Indeed, T cell selection, recruitment, and activation depends on mito-dynamics (44). Moreover, mitochondria integrate a plethora of different intra- and extra-cellular signals both in healthy and cancer cells, thus they behave as central hubs for generating specific “social” signals in both tumoral and TME resident cells, to alter individual cell behavior (pro-inflammatory signals release within the cytosol) or influence target cells. Indeed, mito-dynamics on one side are involved into immune cells activation and migration, on the other are themselves subjects of the ligand-receptor signaling (45, 46), and the induced response has an impact on migrasomes formation and release. Interestingly, released migrasomes can contain mitochondria, thus expanding the kind and quality of signaling delivered by EVs (47).

Mitochondrial activity regulation in TME’s resident cells is of particular importance as it is well known to impact on metabolism, oxidative stress, and apoptosis. Nevertheless, there are still unexplored sides about the role played by mitochondria as potential signaling hubs, in immune cells’ activation, in the modulation of cancer immune evasion, as well as regarding the efficacy of mitochondria-targeted antitumor therapy. We observed that, upon chemoattractant stimulation, migrating T cells produce tubular structures, extending directly from the cell surface, and releasing migrasomes. We speculate that migrasomes released by tumor infiltrating T cells, by acting like “breadcrumbs to be collected” (31), can increase the chemotactic response of recipient cells. Indeed, in the TME, migrasome directionally delivered messages could ameliorate the functionality of different immune cell types, by increasing their proliferation rate, migration, and invasion capabilities. This aspect could be of extreme relevance when considering that all these T cell functions are impaired in TME, inducing an exhausted phenotype. Thus, migrasomes production and modulation could be seen as a new strategy to boost this immune dampening.

## Open questions

The role of exosomes released in the TME has been extensively investigated (14, 48) but the real impact of migrasomes remains still quite obscure in terms of type of message delivered and potential value as biomarkers or therapeutic targets (10).

Another attracting and still poorly investigated hypothesis is related to the possible crosstalk between EVs, such as exosomes and migrasomes, and pro- or anti-tumor cellular pathways like autophagy (49). Indeed, there are evidence supporting a role for exosomes in the regulation of the autophagy induction/execution, especially upon anti-cancer therapy (50), and there is an emerging interest for secretory autophagy (51).

The involvement of migrasomes in the complex regulation of signaling is still largely unknown, even if there are reports about their role in mitochondrial quality-control process through mitocytosis (47), in ischemic brain injury (52), in cerebral amyloid angiopathy (53), in autophagy regulation during miscarriage (54), and in cancer cells undergoing ER-stress, where migrasomes are exploited to release autophagosomes and relieve the stressful condition (55).

Identification of migrasomes, as well as other EVs, in body fluids could be a useful strategy to identify specific markers associated with pathogenic conditions. Also in this case, there is still a lot to understand and standardize prior to an effective bench-to-bedside translation (56, 57).

Another interesting possibility relies on the therapeutic exploitation of different EVs not only as possible drug’s targets but also as drug delivery system (58), even if as of today EV-based therapies and drug delivery, proved to be limited mostly by therapeutic loading technologies and efficiency (59). In this scenario, the possibility to integrate further knowledge about mito-dynamics regulations in TME resident immune cells, the development of drugs specifically targeting mito-dynamics, and their relationships with EVs generation and release could be of main importance to highlight a new way to fight solid tumors. As of today, it has been reported that mitochondria, upon both physiological and stressful conditions (16, 60, 61), can release specific vesicles, namely mitovesicles (34) and mitochondrial-derived vesicles (MDVs) (62), whose biogenesis is somehow related to mito-dynamics and mito-quality control, even if still not fully unveiled (63–66)}. On the other hand, mitochondria transfer through mitoEVs could be of importance in different pathological conditions (16, 67, 68) and mito-dynamics in the recipient cells could be affected upon EVs uptake (67–70). Indeed, as mito-dynamics are fundamental for migrasome generation and release, we could envisage to exploit them to develop CAR-T cells, or other tumor-directed immune cell types, engineered as to release migrasomes along their way into the tumors’ mass, not only to prevent their own exhaustion but also to foster migration and activation of surrounding immune cells.

In conclusion, a better knowledge of cargo composition, release stimuli and mechanisms, selection and effect on the recipient cells are needed to improve the efficacy of a possible EVs-based therapy or to exploit EVs generation *in-situ* as a possible therapeutic approach.

## Data Availability

The original contributions presented in the study are included in the article/supplementary material, further inquiries can be directed to the corresponding author/s.
